# Enhancing diabetes risk prediction through focal active learning and machine learning models

**DOI:** 10.1371/journal.pone.0327120

**Published:** 2025-07-08

**Authors:** Wangyouchen Zhang, Zhenhua Xia, Guoqing Cai, Junhao Wang, Xutao Dong

**Affiliations:** School of Electronic Information and Electrical Engineering, Yangtze University, Jingzhou, China; University of Lahore - Raiwind Road Campus: The University of Lahore, PAKISTAN

## Abstract

To improve the effectiveness of diabetes risk prediction, this study proposes a novel method based on focal active learning strategies combined with machine learning models. Existing machine learning models often suffer from poor performance on imbalanced medical datasets, where minority class instances such as diabetic cases are underrepresented. Our proposed Focal Active Learning method selectively samples informative instances to mitigate this imbalance, leading to better prediction outcomes with fewer labeled samples. The method integrates SHAP (SHapley Additive Explanations) to quantify feature importance and applies attention mechanisms to dynamically adjust feature weights, enhancing model interpretability and performance in predicting diabetes risk. To address the issue of imbalanced classification in diabetes datasets, we employed a clustering-based method to identify representative data points (called foci), and iteratively constructed a smaller labeled dataset (sub-pool) around them using similarity-based sampling. This method aims to overcome common challenges, such as poor performance on minority classes and limited generalization, by enabling more efficient data utilization and reducing labeling costs. The experimental results demonstrated that our approach significantly improved the evaluation metrics for diabetes risk prediction, achieving an accuracy of 97.41% and a recall rate of 94.70%, clearly outperforming traditional models that typically achieve 95% accuracy and 92% recall. Additionally, the model’s generalization ability was further validated on the public PIMA Indians Diabetes DataBase, outperforming traditional models in both accuracy and recall. This approach can enhance early diabetes screening in clinical settings, helping healthcare professionals reduce diagnostic errors and optimize resource allocation.

## Introduction

With the intensification of population aging and lifestyle changes, the prevalence of diabetes is rapidly increasing, particularly in urban areas [1]. Diabetes has become a common chronic disease worldwide, with rising rates observed in both developed countries and emerging economies [2]. This condition contributes to acute and chronic complications that significantly affect patients’ health and quality of life. Therefore, early screening and prediction are critical for comprehensive diabetes management and prevention efforts. Predicting diabetes risk before the onset of symptoms can help reduce complications and enable more effective treatment strategies [3].

However, existing machine learning models often struggle when applied to highly imbalanced datasets, which is common in medical diagnosis tasks like diabetes prediction. These models tend to exhibit high overall accuracy while failing to correctly identify minority class cases, leading to underdiagnosis of at-risk individuals.

In machine learning, imbalanced classification is a well-known challenge, particularly in fields such as fraud detection, medical diagnosis, pollution monitoring, and remote sensing [4]. This imbalance occurs when the distribution of data is skewed, leading to the scarcity of minority class samples. For diabetes datasets, imbalanced classification is common, with far fewer diabetic samples compared to non-diabetic ones. Traditional classification algorithms, which assume balanced data, often struggle to perform well on such datasets, particularly when predicting the minority class. This limitation is further exacerbated by privacy concerns, data-sharing restrictions, and difficulties in acquiring large-scale, high-quality medical datasets. Additionally, diabetes results from a complex interaction of genetic, lifestyle, and environmental factors [5], making simple predictive models insufficient to capture the multifaceted nature of the disease. While more sophisticated models such as deep learning can offer improved performance, they often require significant computational resources and are prone to overfitting, especially when dealing with limited data [6]. Furthermore, healthcare providers have limited resources to collect comprehensive patient data during routine consultations, and many patients are unaware of their health status prior to seeing a doctor [7].

Traditional machine learning algorithms such as Logistic Regression, Support Vector Machines (SVM), and Decision Trees have been widely used for diabetes risk prediction. Logistic Regression, known for its simplicity and interpretability, has been applied in early prediction models, while SVM has been favored for its efficient classification performance. Compared to Logistic Regression, SVM offers improved performance in handling nonlinear and high-dimensional data spaces, making it more suitable for complex clinical datasets*.* However, these methods rely on structured data and often struggle with imbalanced datasets. Ensemble learning methods such as Random Forest and Gradient Boosting Trees have shown better performance by combining multiple base models [8], and their ability to handle high-dimensional data makes them particularly effective for diabetes risk prediction tasks. Mariwan Ahmed Hama Saeed explored diabetes classification and prediction using various machine learning algorithms combined with oversampling techniques [9]. Similarly, Khondokar Oliullah et al. [10] proposed a stacking ensemble learning approach to improve diabetes risk prediction by integrating multiple machine learning models, demonstrating enhanced accuracy and generalization ability in dealing with imbalanced data.

Recent advancements in deep learning have significantly enhanced diabetes risk prediction, enabling models to automatically extract complex features from data and improve prediction accuracy [11]. Convolutional Neural Networks (CNNs) [6] are designed to process spatial data such as medical images, while Recurrent Neural Networks (RNNs) are effective for modeling sequential data like time-series health records. These models have demonstrated impressive capabilities in automatically extracting complex features from data. They are particularly well-suited for processing large amounts of unlabeled data, such as medical images or time-series data, and have proven effective in improving prediction accuracy for complex medical conditions like diabetes. Elias Dritsas et al. [12] introduced a model that combines machine learning and deep learning techniques for diabetes risk prediction, showcasing the advantages of these advanced techniques in processing high-dimensional and unstructured data, ultimately enhancing model performance.

Active learning has emerged as a promising strategy to improve model efficiency by selectively labeling data samples [13]. Recent advancements in active learning and ensemble methods have significantly improved diabetes prediction models, better addressing challenges such as data imbalance and limited labeled data [14–16]. By enabling models to focus on the most informative samples, active learning reduces labeling costs and mitigates data imbalance issues. Wee Boon Feng et al. [17] incorporated active learning methods while working with diabetes datasets, showing that selectively labeling informative samples reduces labeling costs and improves model outcomes. Recent studies highlight the integration of deep active learning techniques in medical image analysis, which not only reduce annotation costs but also enhance model generalization across diverse datasets [18]. However, traditional active learning strategies often emphasize sample uncertainty alone, without considering data distribution or class representation. This can lead to inefficient querying, especially in imbalanced settings where minority-class instances are underrepresented.

In this study, we propose a novel diabetes risk prediction model based on focal active learning, which improves sample selection by focusing on both informativeness and class balance. Focal active learning is a strategy that emphasizes the selection of representative and uncertain samples while giving priority to minority class instances, thereby improving both efficiency and balance in data labeling. The method integrates SHAP (SHapley Additive exPlanations), a model-agnostic interpretability framework that quantifies the contribution of each feature to individual predictions, thereby facilitating better understanding and trust in clinical settings [19]. This enables identification of the most clinically relevant attributes. Recent studies have demonstrated the effectiveness of SHAP in enhancing interpretability for machine learning models in healthcare. For example, SHAP has been successfully applied to tasks such as feature importance evaluation in high-dimensional data [20] and improving the stability of feature attribution methods through advanced algorithms [21].

By integrating clustering techniques, SHAP-based feature importance, and attention mechanisms, this method enhances model interpretability while improving classification performance under imbalanced conditions. Experimental results demonstrate that the proposed approach achieves state-of-the-art performance on the Diabetes-Prediction-Analysis dataset, with further validation conducted on the other public datasets. This study highlights the practical value of focal active learning in real-world healthcare settings, where reducing misdiagnoses and optimizing resource use are key objectives.

The rest of this paper is organized as follows: Dataset Analysis section introduces the datasets and preprocessing techniques. Design Process section presents the proposed methodology in detail. Model Evaluation section explains the key metrics used to evaluate the model’s performance. Results section reports the experimental setup and results. Discussion section provides discussion, limitations, and interpretation. Conclusion section concludes the paper and outlines directions for future research.

## Dataset analysis

The Diabetes-Prediction-Analysis dataset [22] is a public collection of medical and demographic data from patients, along with their diabetes status (positive or negative), a relatively underutilized public dataset obtained from GitHub. The dataset was selected due to its inclusion of modern lifestyle-related variables and its low utilization in existing research, allowing us to validate our method on a relatively novel dataset. The dataset comprises eight features, including age, gender, body mass index (BMI), hypertension, heart disease, smoking history, HbA1c levels, and blood glucose levels, totaling 100,000 data samples. In the Diabetes-Prediction-Analysis dataset, only 8.5% of instances are labeled as diabetic, indicating a severe class imbalance. This poses significant challenges for standard classification algorithms, which tend to prioritize majority-class accuracy over minority-class sensitivity. Given the limited features provided in this dataset, it aligns with the challenge of collecting comprehensive patient data during most clinical visits. Using this dataset allows for model training under conditions of limited sample features, thereby effectively demonstrating the reliability of the proposed method for predicting diabetes classification in imbalanced datasets.

**Fig 1:**
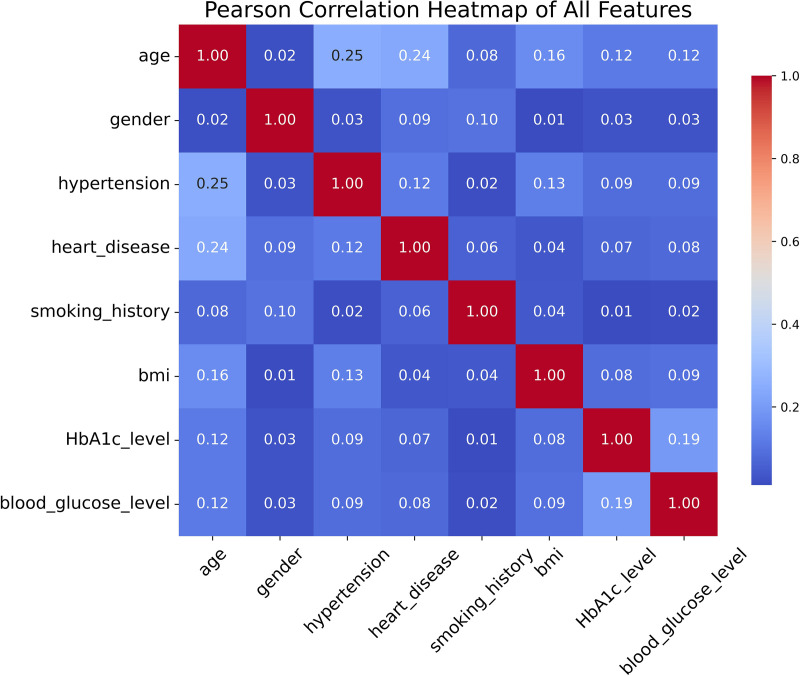
Pearson correlation heatmap of all features in the The Diabetes-Prediction-Analysis dataset.

The dataset underwent a thorough quality check prior to model training. No missing values were found. For models sensitive to feature scale, including SVM, logistic regression, and neural networks, Z-score normalization was applied to continuous variables such as age, BMI, and glucose level. Furthermore, we conducted Pearson correlation analysis on the eight selected features. As shown in Fig 1, all correlation coefficients between variables are relatively low (|r| < 0.3), confirming that there is no significant multicollinearity among the features.

We tested the normality of continuous variables and observed slight deviations, which were expected given the medical nature of the data.

No dimensionality reduction was applied, as correlation analysis revealed no excessive redundancy or collinearity among features. Next, we further analyze the relevant features.

Table 1 displays some characteristics of the dataset used in this experiment. In the gender feature column, 0 represents female and 1 represents male; for hypertension and heart disease feature columns, 0 indicates absence of the condition, while 1 indicates presence. In the smoking history column, 0 denotes never smoked, 1 denotes former smoker, 2 denotes current smoker, and so forth, with the highest level being 4. The HbA1c level indicates glycated hemoglobin percentage; blood glucose level indicates blood sugar levels. Diabetes is labeled as “diabetes,” with a value of 1 for diabetic cases and 0 for non-diabetic cases.

**Table 1:** Diabetes-Prediction-Analysis dataset (8).

gender	age	heart_disease	smoking_history	bmi	HbA1c_level	blood_glucose_level	diabetes	smoking_history
0	36	0	4	23.45	5	155	0	4
1	76	1	4	20.14	4.8	155	0	4
0	20	0	0	27.32	6.6	85	0	0
0	44	0	0	19.31	6.5	200	1	0

Table 2 summarizes the four continuous attributes in the dataset along with their descriptions. “Mean±Std” denotes the mean and standard deviation for each attribute, reflecting both the central tendency and variability. This provides a clearer understanding of the overall data distribution.

**Table 2:** Overview of continuous feature attributes in the Diabetes-Prediction-Analysis dataset.

Attributes	Description	Mean±Std
age	Age in years	46.5441 ± 19.5403
bmi	Body Mass Index (Weight in kg/ (Height in m)2)	28.4241 ± 6.5162
HbA1c_level	For the previous two to three months, average blood glucose (sugar) levels (mmol/l)	5.5643 ± 1.0955
blood_glucose_level	Glycemic Index(mg/dL)	139.6282 ± 42.1659

Next, we analyze the distribution of features within the dataset to provide a basis for selecting initial foci in the subsequent process. This analysis helps identify potential patterns and categories within the dataset, ensuring that the foci can represent the diversity within the dataset and laying a foundation for the subsequent clustering and iterative processes. We present KDE plots for four continuous features (age, BMI, HbA1c, and blood glucose levels), as kernel density estimation is suitable only for continuous variables. KDE is a statistical technique used to estimate the probability density function of a variable, providing a smoothed version of the data distribution. Unlike histograms, KDE avoids binning and provides a more continuous and flexible view of the data, helping to visualize the distribution trend more intuitively. The area under the curves shown in the plots sums to 1, as the KDE estimates the probability density, meaning that the vertical axis represents relative density rather than absolute counts. The shape of the curves illustrates the distribution trend, with peaks indicating higher density and frequency of occurrence near those values.

For instance, the age feature’s distribution may show a peak around middle-aged individuals, which is clinically significant since risk factors for diabetes, such as lifestyle and metabolic changes, tend to rise with age. Similarly, the blood glucose and HbA1c levels might show skewed distributions, with higher concentrations being indicative of a prediabetic or diabetic state, underlining the importance of these features in early detection and risk assessment. These patterns are crucial for identifying the most relevant samples (foci) to optimize our model’s predictive power and performance.

The public dataset used here has limited features, necessitating the model’s reliance on these constrained attributes for predictions. Certain features in the dataset, such as hypertension, heart disease, and smoking history, are discrete binary or multi-value variables and are unevenly distributed.

**Fig 2:**
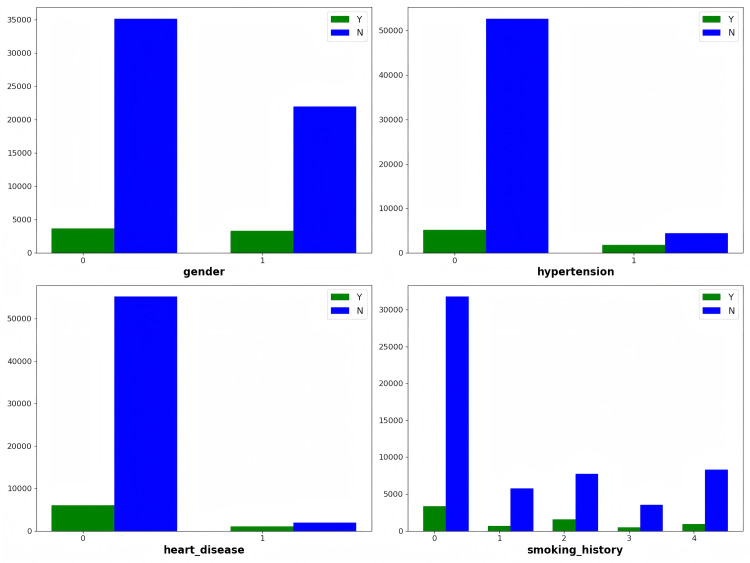
Diabetes-Prediction-Analysis dataset people distribution of 4 attributes in using the bar charts, with green and blue color distributions denoting diabetic (Y) individuals, non-diabetic (N) classes, respectively.

The bar chart in Fig 2 reveals a clear imbalanced classification for features such as gender, hypertension, and heart disease, particularly in the heart disease feature, where the majority of samples are 0, with only a few being 1. Table 2 further indicates that the hypertension and heart disease features have very low means, suggesting that most samples in the dataset are 0 (indicating no hypertension or heart disease). This imbalanced classification may lead the model to favor the majority class during training, neglecting the minority class, which is particularly critical in classification tasks and could result in decreased identification rates for diabetic patients. The model might exhibit high overall accuracy while performing poorly on the minority class (diabetic patients).

**Fig 3:**
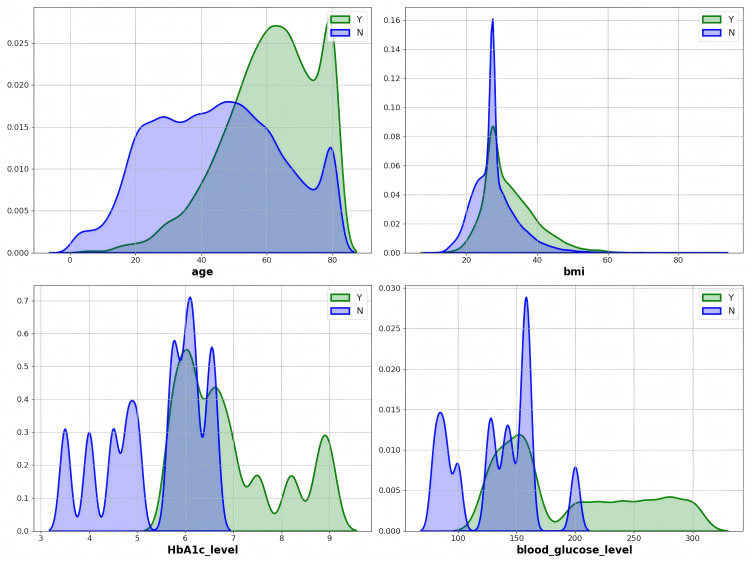
Diabetes-Prediction-Analysis dataset people distribution of 4 attributes in using the KDE graphs, with green and blue color distributions denoting diabetic (Y) individuals, non-diabetic (N) classes, respectively.

Fig 3 illustrates the distribution of continuous variables such as age, BMI, HbA1c level, and blood glucose level. It is evident that these features exhibit significant variability and multimodal distribution. Different groups show substantial differences in these features, especially age and blood glucose level, which display considerable fluctuations, indicating that the model needs to capture the feature patterns across different populations. This multimodal distribution increases the complexity of the model, particularly when there are insufficient features, making it challenging for the model to effectively distinguish between different classes. Moreover, according to Table 2, the features BMI and blood glucose level exhibit large standard deviations, indicating a wide distribution of sample values. Extracting stable patterns from these features poses greater challenges. Additionally, the extensive distribution may imply the presence of outliers or extreme values in the data. If not addressed, these extreme values could adversely affect model training, leading the model to learn from these anomalies rather than general patterns. Handling these outliers is crucial for the stability and predictive performance of the model.

In Table 2, the mean of the HbA1c level is 5.5644, which falls within the normal range (4%−6%). This result suggests that most samples in the dataset do not exhibit prominent diabetes characteristics. If the goal is to predict diabetes, the model may struggle to effectively differentiate between healthy individuals and diabetic patients based solely on this feature. The mean smoking history is close to 1.157, indicating that most samples have a smoking history concentrated in lower ranges, suggesting that many individuals have never smoked or have only mild smoking habits. This implies that this feature may also face imbalanced classification issues, making it easier for the model to overlook the minority class during predictions.

These characteristics present challenges and difficulties in testing whether the model can maintain high predictive performance under limited features. By analyzing the model’s performance with fewer features, we can assess the model’s reliance on critical attributes and its ability to effectively capture key patterns in the data. Furthermore, comparing training results can provide better insights into the model’s generalization ability, the effectiveness of feature learning, and a proper evaluation of the proposed model’s adaptability and generalization capabilities.

Additionally, the model was also tested on the PIMA Indians Diabetes Database, a widely recognized public dataset for diabetes risk prediction. The performance across these datasets demonstrates the robustness and generalization ability of the proposed method.

## Design process

**Fig 4:**
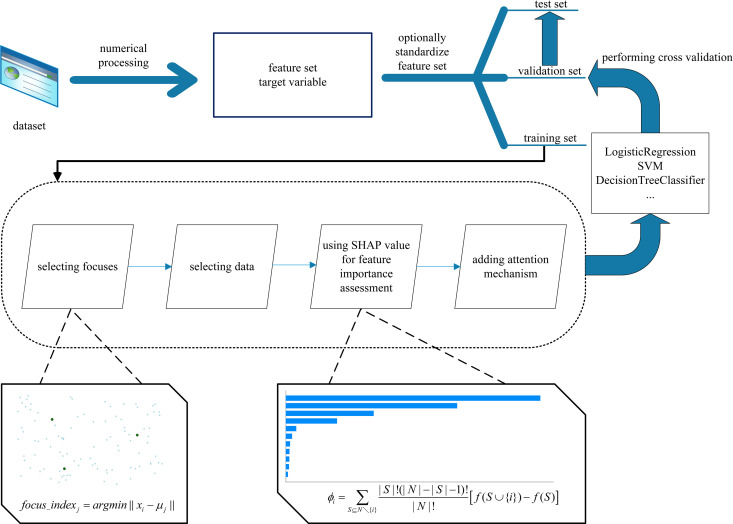
Design of the diabetes risk prediction process.

The proposed diabetes risk prediction process can be divided into two main parts: data preprocessing and model training, as illustrated in Fig 4.

The first part is data preprocessing. In diabetes risk prediction, ensuring data quality is crucial for the accuracy of subsequent analyses and model performance. Data preprocessing is a key step, including cleaning the raw data, handling noise, and performing feature standardization. The feature values in the raw data may have different numerical ranges; therefore, after standardization, all features are scaled to the same range, facilitating further feature evaluation and selection. For example, blood glucose and HbA1c levels often have much larger numerical ranges than categorical features like gender, so normalizing the features helps treat them equally during training.

Next, we divide the dataset into three parts: training dataset (70%), validation dataset (15%), and test dataset (15%).

The second part is model training. In the training dataset, focal selection and sub-pool iterative updates are performed. This part involves three key stages: selecting representative foci through clustering; iteratively constructing subsets based on sample similarity and uncertainty; and applying an attention mechanism to perform feature weighting for enhanced model training. This multi-stage design helps the model focus on the most informative samples, thereby optimizing prediction performance under imbalanced data conditions.

During focal selection, we use the K-Means algorithm to select the initial cluster centers, and similarity is used to select the foci, ensuring that each class in the dataset is appropriately represented. K-Means is a widely used clustering technique, but it assumes spherical clusters and is sensitive to outliers, which may be problematic for datasets with complex feature interactions. We selected K-Means clustering due to its simplicity, scalability, and interpretability. Considering the 8-dimensional data in our dataset and the need for large-scale partitioning, K-Means offers greater efficiency compared to algorithms like DBSCAN or GMM, which are more sensitive to parameter settings or involve higher computational costs. Furthermore, K-Means yields centroid-based clusters, which align well with our goal of selecting representative foci for sub-pool construction.

In the sub-pool construction process, through iterative updates, the number of minority class samples gradually increases, and the class distribution of the dataset becomes more balanced. To ensure computational feasibility, we adopted an iteration termination strategy based on the proportion of labeled samples rather than a fixed number of rounds. Specifically, the active learning process was designed to stop once the labeled sub-pool reached a predefined proportion of the unlabeled dataset. This approach enables adaptive control of iteration steps according to dataset size, ensuring scalability and practicality under standard computational resources. Furthermore, a fixed number of samples was selected during each sub-pool update, which effectively constrained computational overhead and balanced model performance with real-world applicability. During each iteration, we evaluate feature importance using SHAP values and use these assessments to adjust feature weights. SHAP-based feature importance quantifies the contribution of each feature to model predictions, ensuring that critical features—such as glucose levels and age—are given higher importance, enhancing the model’s interpretability and prediction accuracy. This way, the more important features receive greater weight in the dataset, highlighting the influence of key features after weighting.

After completing data preprocessing in the first part, the proportion of labeled minority class samples gradually increases, and the class distribution becomes more balanced. Once the training dataset has undergone focal selection and sub-pool iterative updates, the model proceeds to the next stage. We train the model using various machine learning algorithms, such as logistic regression, support vector machines (SVM), and random forests. We selected a range of models representing varying levels of complexity and interpretability. Logistic Regression was chosen for its transparency and clinical interpretability, especially useful for medical decision-making. Support Vector Machines (SVM) were included due to their strong performance on high-dimensional spaces. Ensemble methods such as Random Forest and XGBoost were used to capture non-linear feature interactions. Neural networks (MLP) and AutoML frameworks (AutoSklearn) were selected to explore machine learning and automated optimization potentials. The selection of these algorithms is based on their proven ability to handle imbalanced datasets, with SVM and Random Forests being particularly effective for classification tasks in medical data. Hyperparameters are tuned, and model performance is evaluated using the validation dataset. For each model, hyperparameter optimization was conducted using grid search combined with five-fold cross-validation. This approach ensures a balanced trade-off between model complexity and performance. For example, the number of estimators and maximum depth were tuned for XGBoost, kernel type and C parameter for SVM, and the number of hidden units and activation function for MLP. During each validation, we use metrics such as precision, recall, and F1-score to assess model performance, adjusting the model based on the validation set to ensure robust performance on imbalanced data. In addition, we conducted ablation experiments to evaluate the contribution of each module by separately removing SHAP-based feature weighting and the attention mechanism, followed by retraining the model. The results showed that removing either module led to a 2–4% decrease in both recall and F1 score, indicating that these components play a critical role in feature importance evaluation and focusing on informative samples.

Finally, we evaluate the model using the test dataset to ensure it can maintain good generalization ability on unseen data. This final step ensures that the model is not overfitting the training data and can effectively predict diabetes risk in real-world scenarios.

### Data preprocessing

Data preprocessing is a crucial step in the machine learning process, as it significantly enhances the reliability of the dataset. The data preprocessing primarily involves the following steps: data cleaning, noise handling, and feature standardization.

Data cleaning aims to remove any invalid or redundant information that may exist within the dataset. During this process, we detected the presence of duplicate values in the dataset, which could negatively impact the quality of model training. Therefore, we employed duplicate detection techniques to eliminate all duplicate samples, ensuring the consistency of the data. No missing values were detected in the dataset. Therefore, no imputation techniques were required.

**Fig 5:**
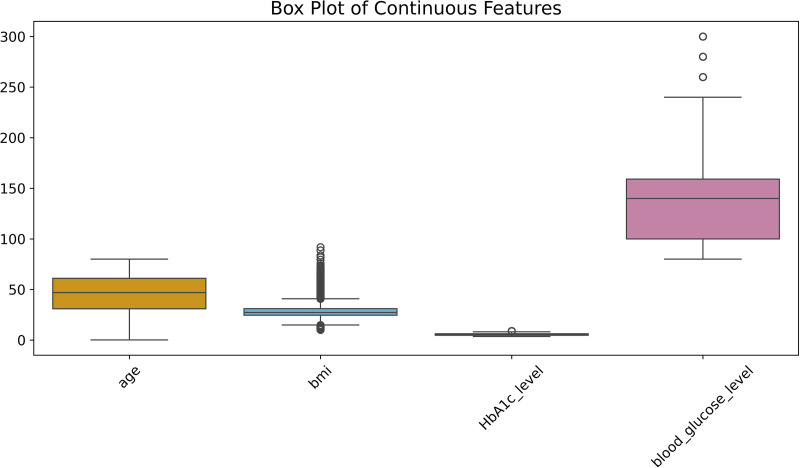
Box plots of continuous features.

Noise refers to the outliers that may exist within the dataset, which can adversely affect the training of the model. To detect these outliers, we utilized the Interquartile Range (IQR) method for continuous variables such as BMI, HbA1c levels, and blood glucose levels. We employed the IQR method to detect and remove outliers due to its robustness against skewed distributions and its effectiveness in identifying extreme values in medical datasets. The IQR method identifies extreme values by calculating the quartiles for each variable and detecting values that exceed 1.5 times the IQR range. After processing, we removed the corresponding outliers to enhance the stability of the dataset and improve the predictive accuracy of the model. To further illustrate the distribution and potential outliers of the key numerical features, we plotted a box-and-whisker plot for four continuous variables—age, BMI, HbA1c level, and blood glucose level (Fig 5). Compared to the IQR method alone, the box plot offers an intuitive visualization of quartiles, spread, and extreme values, thereby enhancing the interpretability of the preprocessing step and supporting decisions related to outlier removal.

Different features may have varying numerical ranges and units, making it necessary to standardize these parameters before model training to ensure that all parameter values are within a reasonable range. Most machine learning algorithms require numerical input; thus, we converted all feature columns to numerical types, simplifying the data preprocessing process and improving data consistency and processing efficiency.

Next, we partitioned the dataset into feature set X and target variable Y. In the feature set, certain features exhibited significant variations in numerical ranges and had distributions that approximated a normal distribution, leading us to standardize these feature columns. Here, we chose Z-score normalization over Min-Max scaling because Z-score better reflects the distribution characteristics and is more suitable for scale-sensitive models, such as SVM and MLP. The standardization formula is given by:


$$x=\frac{x^{\prime }-\gamma }{\sigma }$$ (1)

where x is the standardized value, $x^{\prime }$ is the original data value, γ is the mean of the data, and σ is the standard deviation of the data.

Categorical variables such as gender and smoking history were encoded numerically using label encoding. No dimensionality reduction or feature elimination was applied, as preliminary analysis showed no strong multicollinearity or redundancy among features (Pearson’s |r| > 0.8).

After completing the data cleaning, noise handling, and feature standardization, we ensured the consistency and reliability of the data, providing a solid foundation for subsequent model training. We then divided the entire dataset into a training dataset (70%), a validation dataset (15%), and a test dataset (15%). Given the extreme class imbalance (with diabetic patients comprising only 8.5%), class weights were introduced when training models such as SVM and logistic regression, which are highly sensitive to imbalanced data, and ensured the sub-pool construction in focal active learning sampled more from minority class regions.

When validating the effect of preprocessing, we found that after applying IQR outlier removal and data normalization, the F1 scores for scale-sensitive models such as SVM and MLP increased by an average of 2.7%, indicating that the preprocessing steps had a positive impact on model performance.

Once the data preprocessing is completed, the next step is model training on the training dataset.

### Model training

#### Focal selection.

For large datasets, the number of clustering centers (initial foci) can be automatically selected using clustering algorithms. Specifically, we employed the K-Means clustering algorithm to extract initial foci from unlabeled samples. K-Means was selected as the clustering method for initial focal selection due to its simplicity and scalability. Given the high-dimensional nature of our dataset and the large sample size, K-Means offered a computationally efficient solution with clear centroid representation. The K-Means algorithm partitions the dataset into multiple clusters based on sample features and calculates the centroid of each cluster as its representative point, or focus. These foci will be used for subsequent iterative updates and sub-pool construction.

The number of clustering centers *k* is chosen to be in the vicinity of $\sqrt{\frac{n}{2}}$, where *n* represents the number of data points in the dataset. Each focus represents the center of a cluster, and as the algorithm iterates, these foci gradually adjust until they stabilize at the optimal representation within the data distribution. By continually refining the positions of these foci, we can converge to a stable clustering structure, effectively capturing the complex patterns within the data.

Next, we will describe how to iteratively update these foci using the K-Means algorithm. K-Means has been extensively researched and optimized, and many programming languages and data analysis tools provide convenient implementations that can efficiently capture the diversity and distribution characteristics of the data. During the process of selecting foci from the training set, the data points are assigned to different clusters such that points within each cluster are similar in some sense, while maximizing the differences between clusters. By selecting the centroid of each cluster as a focus, we ensure that the chosen samples are representative of the entire dataset.

Given a training dataset $=\{x_{1},x_{2},...,x_{n}\}$, we initialize k cluster centers (initial foci) $\mu_{1},\mu_{2},...,\mu_{k}\;$, where k=200 in this study. For each point $x_{i}$ in cluster $c_{j}$, the cosine similarity between the point and the cluster center $\mu_{j}$ is calculated as follows:


$$\begin{array}{c} CosineSimilarity({\vec{x}}_{i},{\vec{\mu }}_{j})=\frac{{\vec{x}}_{i}\cdot {\vec{\mu }}_{j}}{\left|\right|{\vec{x}}_{i}\left||\times |\right|{\vec{\mu }}_{j}\left|\right|} \end{array}$$ (2)

Where ${\vec{x}}_{i}$ represents the feature vector of point $x_{i}$ and ${\vec{\mu }}_{i}$ represents the feature vector of cluster center $\mu_{j}$ The dot product ${\vec{x}}_{i}\cdot {\vec{\mu }}_{j}$ is used to calculate the angle between the two vectors, and $\left||{\vec{x}}_{i}|\right|$ and $\left||{\vec{\mu }}_{j}|\right|$ are the norms of the vectors, respectively. The value of cosine similarity ranges from [−1,11], with values closer to 1 indicating greater similarity between the two samples.

Each point $x_{i}$ is assigned to the cluster center to which it is most similar:


$$\begin{array}{c} c_{i}=arg\underset{j}{\max}\;\;CosineSimilarity({\vec{x}}_{i},{\vec{\mu }}_{j}) \end{array}$$ (3)

For each cluster $c_{j}$, the cluster center $\mu_{j}$ is updated as the mean of all samples within that cluster:


$$\mu_{j}=\frac{1}{|c_{j}|}\sum_{x_{i}\in c_{j}}\;x_{i}$$ (4)

Where $\left|c_{j}\right|$ is the number of data points in cluster $c_{j}$.

Once clustering is complete, for each cluster $c_{j}$, the sample most similar to the cluster center is selected as the representative (focus) of that cluster:


$$\begin{array}{c} focus\_index_{j}=arg\underset{i}{max\;}\;CosineSimilarity({\vec{x}}_{i},{\vec{\mu }}_{j}) \end{array}$$ (5)

Where $focus\_index_{j}$ is the index of the selected focus in the dataset. These foci represent the diversity and different patterns within the dataset. They will be used for the subsequent sub-pool construction and iterative updates.

**Fig 6:**
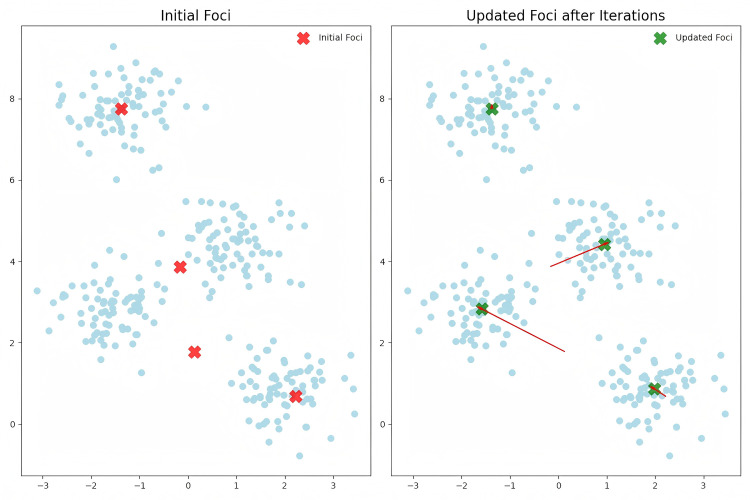
Example of the iterative update process of the focus after dimensionality reduction via t-SNE.

To visually illustrate the iterative process of focal point updates, this study employs t-distributed Stochastic Neighbor Embedding (t-SNE) to reduce the dimensionality of the eight-dimensional features, projecting the data into a two-dimensional space for visualization. It should be noted that dimensionality reduction is used solely for visualization purposes; the actual clustering and focal point updating process takes place in the original eight-dimensional feature space. t-SNE preserves the local structure between data points in the high-dimensional space, allowing the points in Fig 6 to more intuitively reflect their relative proximity in two-dimensional space. This enables us to observe how the foci move from their initial positions to their final locations during the clustering process.

The features in our dataset (e.g., age, BMI, blood glucose levels, etc.) have specific numerical ranges, but after dimensionality reduction using t-SNE, these eight-dimensional features are compressed into two dimensions. t-SNE displays the data by optimizing the relative distances between points, and the coordinates themselves may undergo scaling or translation. Therefore, the x- and y-axis values in the t-SNE plot have no direct relationship with the feature values in the original dataset; they are merely used to visually present the relative positions of the data points in a 2D plane. The coordinate range of −3–8 is adaptively generated by the t-SNE algorithm, with the primary goal being to reasonably display the distribution of data points on the graph.

Fig 6 uses t-SNE dimensionality reduction to display the distribution of the initial foci and the foci after iterative updates. The red “X” symbols represent the initial foci, which are distributed across different regions of the data, representing different patterns or features within the dataset. As the iterations progress, the foci gradually move closer to the green “X” symbols, indicating the gradual convergence of foci during the clustering process. Eventually, the foci cease to move significantly, signaling that the iterative process has stabilized. Through this visualization, we can intuitively observe how the iterative updates affect the selection and adjustment of representative samples in the dataset.

#### Sub-pool construction.

Based on the stabilized foci (labeled samples), the sub-pool is constructed by selecting samples from the unlabeled dataset that are similar to the foci, using similarity measures. The similarity between each unlabeled sample $x_{i}$ and focal point $\mu_{j}$ is calculated according to the second formula.

During the sub-pool construction and sample selection process, uncertainty is assessed for each unlabeled sample based on the predicted probabilities. Priority is given to samples with high prediction uncertainty for labeling. The uncertainty of a sample $x_{i}$ is calculated as follows:


$$uncertaintx_{i}=-\underset{c}{\max}\;P(y=c|x_{i})$$ (6)

Where $uncertaintx_{i}$ represents the uncertainty of sample $x_{i}$, $P(y=c|x_{i})$ is the model’s predicted probability that sample $x_{i}$ belongs to class c and $\max_{c}$ represents the most confident predicted class for $x_{i}$. Prioritizing samples with higher uncertainty helps improve the model’s learning efficiency.

In imbalanced datasets, minority class samples are often scarce, so the following method is used to ensure that minority class samples are prioritized for labeling:


$$minority_{class}=arg\underset{c}{\min}\;\sum_{i}\;\delta \left(y_{i}=c\right)$$ (7)

Where minority_class represents the label of the minority class, and $\delta \left(y_{i}=c\right)$ is an indicator function that equals 1 if $y_{i}=c$, otherwise 0. This is used to calculate the number of samples for each class.

To simultaneously account for similarity, uncertainty, and minority class prioritization, a combined score is computed for each sample to determine which samples should be labeled and added to the sub-pool:


$$\begin{array}{c} combined\_score_{i}=\alpha \cdot CosineSimilarity\left({\vec{x}}_{i},{\vec{\mu }}_{j}\right)+\beta \cdot uncertainty_{i}+\gamma \cdot \delta (y_{i}=minority\_class) \end{array}$$ (8)

Where $combined\_score_{i}$ represents the comprehensive score for sample $x_{i}$, used to prioritize its selection. $\delta (y_{i}=minority\_class)$ equals 1 if the sample $y_{i}$ belongs to the minority class, otherwise 0. α,β,γ are weight parameters used to balance the influence of similarity, uncertainty, and minority class prioritization.

#### SHAP feature importance evaluation and attention mechanism weighting.

After incorporating the selected samples into the sub-pool, we assess the importance of the features of the samples in the current sub-pool using SHAP (SHapley Additive exPlanations) values. Additionally, an attention mechanism is applied to weight these features. The SHAP value is calculated as follows:


$$\begin{array}{c} \phi_{i}=\sum_{S\subseteq N\setminus \{i\}}\;\frac{\left|S\right|!(\left|N\right|-\left|S\right|-1)!}{\left|N\right|!}\left[f\left(S\cup \left\{i\right\}\right)-f\left(S\right)\right] \end{array}$$ (9)

Where $\phi_{i}$ represents the SHAP value of feature i, which reflects the contribution of that feature to the model’s prediction. S represents a subset of features, N is the set of all features, and f(S) is the model’s prediction output based on the subset S. By dynamically evaluating the contribution of each feature using SHAP values, we track the importance of features as new labeled samples are added during each iterative update. This reflects the model’s deepening understanding of different features over time. As the iterations proceed, the SHAP values for the features gradually stabilize, and the weighting evaluation of key features becomes more precise, particularly for important features of minority class samples.

The attention-weighted value for each feature is then calculated based on the SHAP values:


$$\begin{array}{c} weighted\_feature_{i,j}=featur\;e_{i,j}\times importanc\;e_{j} \end{array}$$ (10)

Where $weighted\_feature_{i,j}$ is the weighted value of the i−th sample’s j−th feature, $feature_{i,j}$ is the original feature value, and $importance_{j}$ is the importance of feature j, as determined by the SHAP values. This approach ensures that the feature weights are dynamically adjusted based on the labeled samples, with each iteration focusing on the most representative and significant features.

**Fig 7:**
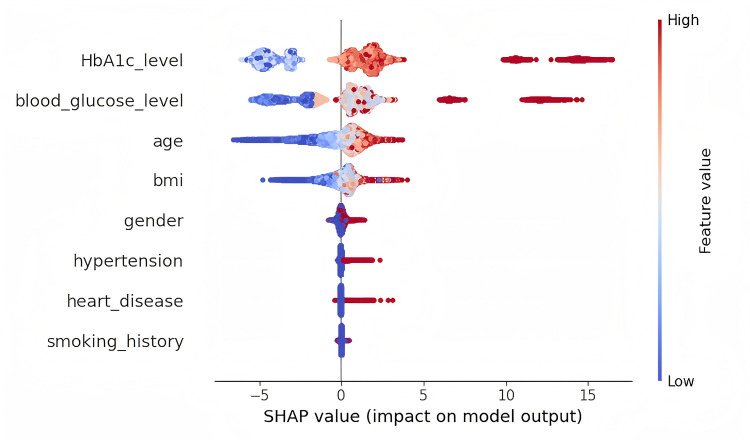
SHAP value summary graph.

**Fig 8:**
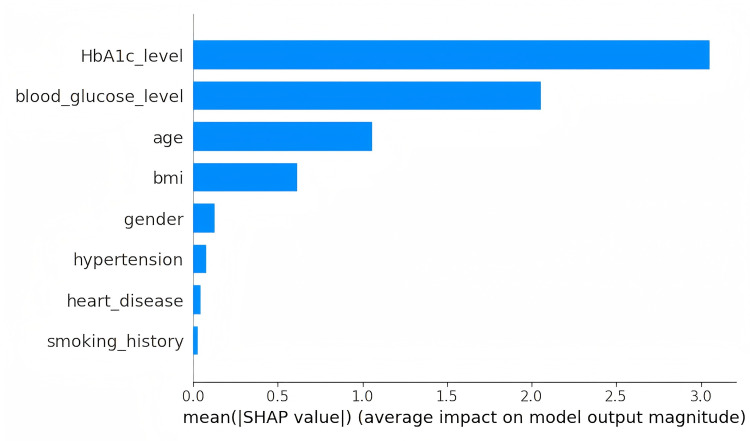
SHAP value feature importance graph.

Figs 7 and 8 show the results of the feature importance assessment after multiple rounds of iterative updates. The SHAP values in these figures reflect the long-term impact of the features on the prediction task. Figs 7 and 8 respectively display the SHAP summary plot and the feature importance plot generated from the Diabetes-Prediction-Analysis dataset after multiple iterations have converged.

The SHAP method reveals the interactions and nonlinear relationships between features by calculating each feature’s contribution to the prediction outcome. This allows us to gain a comprehensive understanding of how each feature affects the target variable. In Fig 7, the x-axis represents the SHAP values, with larger values indicating a greater influence of the feature on the prediction task. Positive SHAP values indicate that increasing the feature value increases the output, while negative values indicate the opposite. The position of the features on the y-axis reflects their ranking in the dataset, usually sorted by importance, with the most influential feature at the top. The color represents the magnitude of the feature values, with red indicating high feature values and blue indicating low values. Each point on the plot represents a specific feature value and its corresponding SHAP value in the dataset.

Fig 8 displays the ranking of feature importance, with the most influential feature for the prediction task at the top. SHAP values fairly distribute the contribution of each feature, ensuring consistency and fairness in feature evaluation. This method is not affected by feature order or algorithm type, providing consistent explanations for all types of prediction tasks. This helps us understand the distribution of feature values and their impact on the prediction outcome. Subsequently, the attention mechanism based on SHAP values is used to weight the features. By emphasizing the most important features, the information in the data can be more effectively expressed, improving the accuracy of predictions for the target variable.

#### Sub-pool iterative updates.

To ensure computational feasibility, we adopted an adaptive stopping criterion based on the proportion of labeled samples rather than a fixed number of iterations. Specifically, the active learning process was designed to terminate once the labeled sub-pool reached 20% of the total unlabeled dataset. Each sub-pool update involved selecting a fixed batch of 400 samples with the highest combined scores, which were calculated based on cosine similarity, prediction uncertainty, and minority class prioritization. This controlled setting ensured that the model focused on the most informative and representative instances, while remaining scalable under standard computational resources.

The sub-pool was iteratively updated based on a sample ranking mechanism. In each iteration, unlabeled samples were scored using a combined metric that integrates the three factors above. Samples with the highest scores were selected and added to the sub-pool. This strategy ensured both targeted sampling and progressive rebalancing of class distributions during the labeling process.

After each iteration, the labeled samples in the sub-pool are fed back into the overall training dataset, while they are removed from the pool of unlabeled samples. As the sub-pool undergoes successive iterations, the total number of unlabeled samples in the training set gradually decreases, and more minority class samples are progressively labeled, helping to address the imbalanced classification problem in the dataset. As more labeled samples are fed back into the dataset, the overall data distribution progressively aligns with the true distribution, improving the accuracy and representativeness of the sub-pool sample selection.

This feedback mechanism effectively drives the next round of sub-pool iterative updates, ensuring that each iteration increasingly foci on the most difficult-to-classify samples in the unlabeled dataset. This, in turn, enhances the learning capacity and generalization performance of the method.

Through the iterative updates of the sub-pool, the proportion of labeled minority class samples steadily increases, resulting in a more balanced class distribution in the dataset. This shift improves the representation of minority class samples, effectively reducing biases caused by data imbalance. Simultaneously, with the dynamic evaluation of feature importance using SHAP values, the weights of key features in the dataset are adjusted with greater precisio00on. The weighted features effectively capture critical information within complex patterns, ensuring that important features of minority class samples are better expressed. We compared SHAP with permutation importance and recursive feature elimination (RFE) and found that SHAP consistently highlighted clinically relevant features (e.g., HbA1c, glucose). It also resulted in a slight but consistent improvement in F1-score (approximately +1.2%) across models, supporting its use in our pipeline.

After the final iteration, a sufficient number of minority class samples have been labeled, significantly enhancing the overall data balance and improving the uniformity of the class distribution. The weighted features allow the dataset to more comprehensively reflect the patterns present in the actual data, laying a more solid foundation for subsequent model training.

#### Combined with machine learning.

Following the completion of the sub-pool iterative updates, the following machine learning models are used for training: linear regression, logistic regression, support vector machines (SVM), decision trees, random forests, gradient boosting trees (such as XGBoost, LightGBM, CatBoost), naive Bayes, and neural networks.

Support Vector Machines (SVM) construct an optimal hyperplane to maximize the margin between classes, making them effective in high-dimensional spaces and particularly useful for imbalanced datasets. Compared to logistic regression, SVM offers improved performance in non-linear classification tasks due to its use of kernel functions. XGBoost is an efficient gradient boosting framework that builds decision trees sequentially to minimize residual errors. Compared to random forests, XGBoost incorporates regularization techniques and advanced pruning strategies, making it more robust and less prone to overfitting.

During the model tuning phase, 5-fold cross-validation is employed to evaluate model performance and fine-tune hyperparameters. In each iteration, the validation dataset is used to assess performance metrics such as accuracy, recall, and F1-score. This cross-validation approach allows us to obtain more stable performance results across different hyperparameter configurations, ensuring the model’s ability to generalize to unseen data.

After model tuning and cross-validation, the test dataset is used for final evaluation. Performance metrics such as accuracy, recall, and F1-score on the test dataset provide a comprehensive assessment of the model’s overall performance, ensuring that it is robust and performs well in real-world applications. The final test results will demonstrate the model’s stability and predictive capability, particularly in handling imbalanced datasets.

### Model evaluation

In this study, we use accuracy, precision, recall, and F1-score as the key metrics to evaluate the quality of the model.

Accuracy refers to the proportion of correctly predicted samples out of the total number of samples. It is calculated as:


$$\begin{array}{c} Accuracy=\frac{TP+TN}{TP+TN+FP+FN} \end{array}$$ (11)

In the formula, TP (True Positive) is the number of instances where the model correctly predicts a positive class as positive. TN (True Negative) is the number of instances where a negative class is correctly predicted as negative. FP (False Positive) represents the number of incorrect predictions where a negative instance is wrongly classified as positive. FN (False Negative) is the number of incorrect predictions where a positive instance is wrongly classified as negative.

In this study, the positive class refers to diabetic patients, while the negative class refers to non-diabetic individuals.

Precision measures the proportion of samples predicted as positive that are actually positive. It reflects the correctness of the model in predicting positive instances:


$$\begin{array}{c} Precision=\frac{TP}{TP+FP} \end{array}$$ (12)

Recall (also known as sensitivity) refers to the proportion of actual positive samples that are correctly predicted by the model. It is calculated as:


$$Recall=\frac{TP}{TP+FN}$$ (13)

F1-score is the harmonic mean of Precision and Recall:


$$\begin{array}{c} F1-score=2\times \frac{Precision\times Recall}{Precision+Recall} \end{array}$$ (14)

The best F1-score is 1, while the worst is 0. The F1-score is particularly useful when the dataset is imbalanced, as it balances the trade-off between Precision and Recall.

## Results

To implement the data processing pipeline and active learning framework, we utilized Python. Key Python packages included scikit-learn for machine learning models and preprocessing, Pandas for data manipulation, NumPy for numerical operations, and Matplotlib for data visualization. Additionally, the AutoSklearn framework was used to automate model selection and hyperparameter tuning. This setup ensured scalability and flexibility throughout the experimentation process.

In this experiment, during the model training phase, the number of initial foci was set to 200, the number of samples added to the sub-pool in each iteration was set to twice the initial number of foci, and the final sub-pool size was set to 20% of the total amount of unlabeled data. These three parameters were fine-tuned using cross-validation. The final combination of parameters was the one that performed the best on the validation set, ensuring that the model maintained sample balance while also achieving good generalization ability.

Through this active learning mechanism, the sub-pool was continually adjusted, and the model weights were updated. Subsequently, the final model was evaluated on the test set, and the best performance results for each machine learning classification method were calculated, including accuracy, precision, recall, and F1-score.

**Fig 9:**
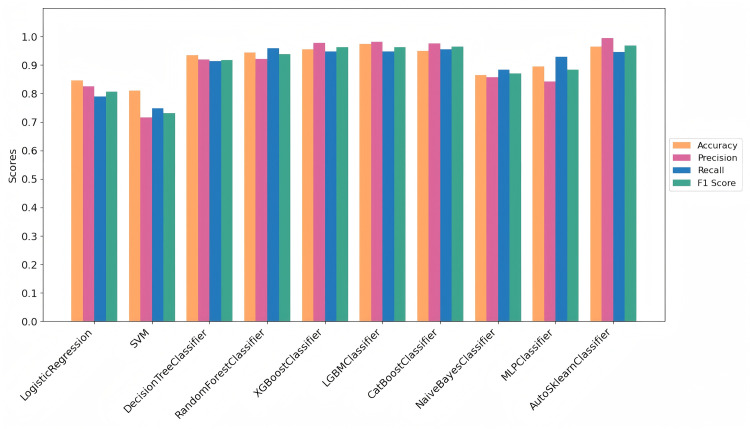
Performance Metrics by Methods in the Diabetes-Prediction-Analysis Dataset.

Table 3 and Fig 9 present the best results for each machine learning classification method. The metrics—Accuracy, Precision, Recall, and F1 Score—are calculated for each model. Table 2 specifically highlights the best results for diabetes risk prediction, with recall being particularly important due to the high cost of missed diagnoses (i.e., false negatives) in the application scenario. Based on the results shown in Fig 9, we can visually observe and conclude that AutoSklearn, XGBoost, and LGBM achieve the top three highest accuracy scores.

**Table 3:** Comparison of Machine Learning Methods in the Diabetes-Prediction-Analysis dataset. We mark the top three results in bold.

Method	Accuracy	Precision	Recall	F1-score
LogisticRegression	0.8456	0.8261	0.7890	0.8061
SVM	0.8102	0.7156	0.7482	0.7315
DecisionTreeClassifier	0.9347	0.9198	0.9145	0.9180
RandomForestClassifier	0.9441	0.9210	0.9586	0.9388
**XGBoostClassifier**	**0.9551**	**0.9782**	**0.9483**	**0.9625**
**LGBMClassifier**	**0.9741**	**0.9806**	**0.9470**	**0.9632**
CatBoostClassifier	0.9492	0.9765	0.9543	0.9648
NaiveBayesClassifier	0.8654	0.8578	0.8845	0.8707
MLPClassifier	0.8945	0.8420	0.9296	0.8836
**AutoSklearnClassifier**	**0.9650**	**0.9941**	**0.9456**	**0.9690**

This comparison highlights the effectiveness of the proposed approach and its ability to balance model performance with class representation, especially in the context of imbalanced datasets.

**Fig 10:**
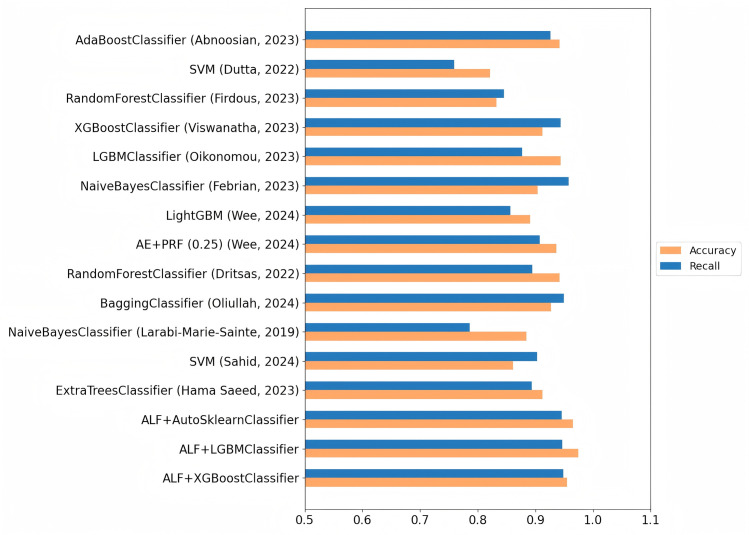
Comparison of accuracy and recall between different algorithms.

**Fig 11:**
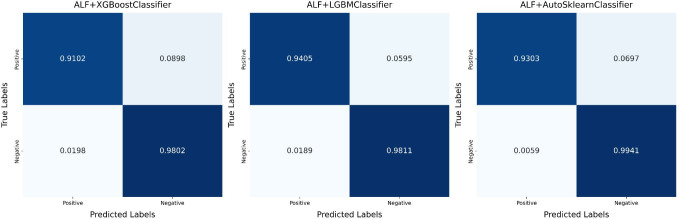
The confusion matrix corresponding to the best results in this study. In the matrix, TP, TN, FP, and FN have all been naturally turned into numbers between 0 and 1.

Table 4 presents the best results obtained in this study and compares them with other research works [6,8–10,12,17,23–28]. Using the same dataset, models were constructed based on the methods proposed in these studies. For diabetes risk prediction, the evaluation metrics include accuracy, precision, recall, and F1-score. The results of this study are more accurate compared to those of previous studies. Fig 10 provides an intuitive comparison of accuracy and recall between the different algorithms applied to the same dataset in this study, where ALF represents Active Learning with Focal, the method proposed in this paper. Fig 11 shows the confusion matrix corresponding to the best results in this study.

**Table 4:** Comparison of algorithms from other papers. We mark the top three results in bold.

Algorithm	Accuracy	Precision	Recall	F1-score
**ALF + XGBoostClassifier**	**0.9552**	**0.9782**	**0.9483**	**0.9625**
**ALF+LGBMClassifier**	**0.9741**	**0.9806**	**0.9470**	**0.9632**
**ALF+AutoSklearnClassifier**	**0.9653**	**0.9941**	**0.9456**	**0.9690**
ExtraTreesClassifier(Hama Saeed,2023)	0.9123	0.9421	0.8936	0.9172
SVM(Sahid,2024)	0.8612	0.7683	0.9030	0.8301
NaiveBayesClassifier(Larabi-Marie-Sainte,2019)	0.8847	0.9472	0.7865	0.8594
BaggingClassifier(Oliullah,2024)	0.9274	0.9140	0.9498	0.9316
RandomForestClassifier(Dritsas,2022)	0.9421	0.9789	0.8943	0.9344
AE + PRF(0.25)(Wee,2024)	0.9362	0.9693	0.9078	0.9375
LightGBM(Wee,2024)	0.8913	0.9496	0.8564	0.9003
NaiveBayesClassifier(Febrian,2023)	0.9037	0.8634	0.9581	0.9082
LGBMClassifier(Oikonomou,2023)	0.9435	0.9277	0.8769	0.9016
XGBoostClassifier(Viswanatha,2023)	0.9118	0.9019	0.9442	0.9226
RandomForestClassifier(Firdous,2022)	0.8327	0.5633	0.8457	0.6748
SVM(Dutta,2022)	0.8214	0.8511	0.7591	0.8024
AdaBoostClassifier(Abnoosian,2023)	0.9417	0.9779	0.9265	0.9514

Hama Saeed et al. used the Extra Trees classifier to handle the classification task for Type 2 diabetes. Their method achieved an accuracy of 91.23% on the dataset used in this study; however, the recall was only 89.36%, which is significantly lower than the recall achieved by the method proposed in this paper. Wee et al. combined an autoencoder (AE) and a probabilistic random forest (PRF) in a hybrid model to handle the diabetes dataset. The autoencoder was used for feature extraction, while the PRF performed the final classification task. The accuracy of their models is 93.62%, which is high in all methods, but precision and recall do not meet expectations. In addition, the hybrid model improves calculation complexity, resulting in longer training. Additionally, the hybrid model increased computational complexity, leading to longer training times. The deep structure of AE and the complexity of PRF also negatively impacted the model’s interpretability, which may limit its application in practical healthcare settings. Wee et al. also explored the LightGBM method. After setting the corresponding parameters for training on the dataset used in this study, the recall was only 85.64%, performing poorly. Viswanatha et al. employed the XGBoostClassifier, which improves training efficiency and performance through parallel computing and specific algorithm optimizations such as shrinkage and regularization. However, the performance of XGBoost is highly dependent on parameter settings. While the default parameters often work well, achieving the best performance may require extensive parameter tuning, increasing the complexity of model development. In their study, the XGBoost model applied to the Diabetes-Prediction-Analysis dataset achieved a high recall of 94.42%, but the accuracy was only 91.18%, which is insufficient for practical medical needs. We also built the model used by Abnoosian et al. with AdaBoostClassifier to train the dataset. However, AdaBoost is sensitive to noise in the data, which can increase the weight of misclassified samples, leading to overfitting when the dataset contains noise or outliers, thus affecting the prediction performance. The actual training results of AdaBoostClassifier showed an accuracy of 94.14%, with a recall of 92.65%.

**Fig 12:**
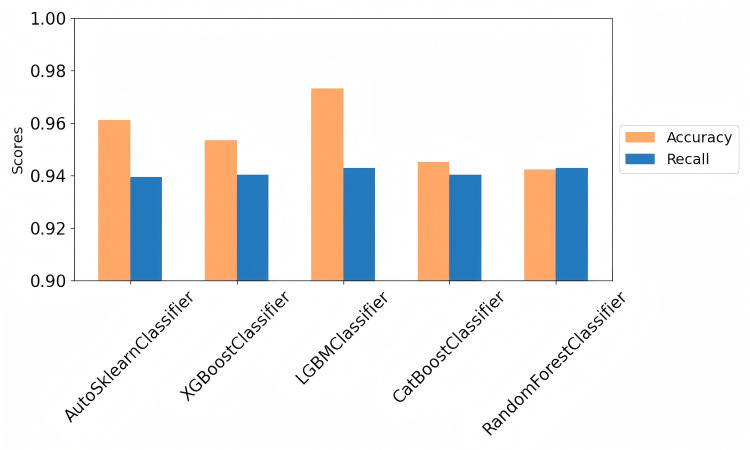
Comparison of the mean accuracy and recall of different classifiers in cross validation.

The accuracy and recall were validated through 5-fold cross-validation. For both the test and training samples, we selected a 1:4 ratio between non-diabetic and diabetic cases. Table 5 presents the analysis results for the following machine learning models used in this study: CatBoostClassifier, AutoSklearnClassifier, RandomForestClassifier, XGBClassifier, and LGBMClassifier. Fig 12 shows the mean comparison of accuracy and recall for the five classifiers during cross-validation.

**Table 5:** Mean and standard deviation of accuracy and recall of different classifiers in cross validation. Mean: average of various metrics; SD (×10 − 3): standard deviation of various metrics.

Method	Accuracy		Recall	
	Mean	SD	Mean	SD
AutoSklearnClassifier	0.9610	1.6370	0.9395	7.5287
XGBoostClassifier	0.9533	3.036	0.9403	8.3515
LGBMClassifier	0.9732	0.2650	0.9428	4.8842
CatBoostClassifier	0.9452	8.0240	0.9403	8.8051
RandomForestClassifier	0.9423	2.7760	0.9429	13.2570

It is evident that when the models were combined with the LGBM classifier, the metrics remained nearly consistent across all five iterations. Therefore, the LGBM model, when integrated with focal active learning, demonstrated stability, making it particularly reliable when detecting diabetes.

To further verify the generalization ability of the proposed model, we validated the model on the public Pima Indians Diabetes Database, a widely used benchmark dataset for diabetes prediction [29]. The experimental results were compared with those obtained using simpler models, such as Logistic Regression and Decision Trees, without incorporating the proposed active learning strategy. The comparison demonstrated that the proposed method significantly outperformed these baseline models in terms of accuracy and recall, highlighting its effectiveness in handling imbalanced and complex datasets..

The Pima Indians Diabetes Database is composed of structured diagnostic data from Pima Indian females aged 21 and above. Based on the knowledge and expertise of medical professionals, the dataset includes several interpretable features such as BMI, insulin level, and the number of pregnancies, providing meaningful predictors for diabetes classification. We applied focal active learning combined with three advanced classifiers—RandomForestClassifier, XGBClassifier, and LGBMClassifier—to train the model on this dataset. Additionally, we included two simpler models, Logistic Regression and Decision Tree, which were not integrated with the proposed active learning strategy, as baseline comparisons.

**Fig 13:**
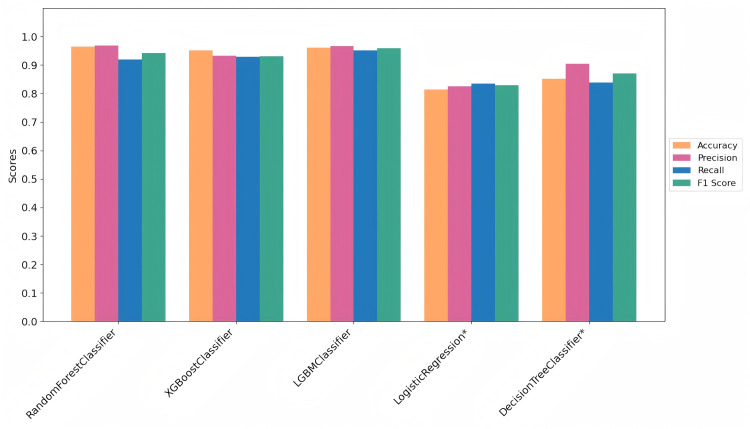
Performance Metrics by Methods in the Pima Indians Diabetes Database.

**Fig 14:**
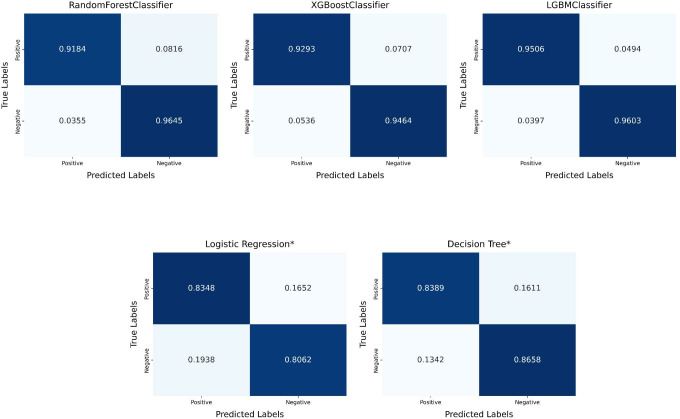
The confusion matrices obtained according to the model in the Pima Indians Diabetes Database.

Table 6 and Fig 13 present the results obtained from these five machine learning classifiers on the Pima Indians Diabetes Database. It was observed that the combination of focal active learning and the LGBMClassifier achieved the best performance, with an accuracy of 96.07% and a recall of 95.06%. Fig 14 represents the confusion matrix obtained in the Pima Indians Diabetes Database. Similarly, the simple model that does not actively learn shows lower performance, and once again verified the effectiveness of the diabetes predictive strategy.

**Table 6:** Comparison of Machine Learning Methods in the Pima Indians Diabetes Database.

Method	Accuracy	Precision	Recall	F1-score
RandomForestClassifier	0.9643	0.9674	0.9184	0.9423
XGBoostClassifier	0.9504	0.9321	0.9293	0.9307
LGBMClassifier	0.9607	0.9658	0.9506	0.9581
Logistic Regression*	0.8137	0.8247	0.8348	0.8297
Decision Tree*	0.8524	0.9047	0.8389	0.8706

## Discussion

When detecting diabetes, the risk of misclassifying labels remains substantial. Early identification of high-risk individuals is critical for implementing preventive measures to mitigate the onset of diabetes and its complications. While such early interventions may incur some additional medical expenses, these costs are relatively low compared to the long-term healthcare burden caused by untreated or late-stage diabetes. For instance, if an individual is incorrectly classified as being at high risk for diabetes, medical institutions can recommend additional diagnostic tests and monitoring. If follow-up results confirm that the individual is not at risk, medical intervention can cease, allowing the person to continue with their life as usual. This approach balances the cost of unnecessary interventions with the benefit of reducing long-term healthcare expenses.

When detecting diabetes, the risk of misclassifying labels remains substantial. Early identification of high-risk individuals is critical for implementing preventive measures to mitigate the onset of diabetes and its complications. While such early interventions may incur some additional medical expenses, these costs are relatively low compared to the long-term healthcare burden caused by untreated or late-stage diabetes. For instance, if an individual is incorrectly classified as being at high risk for diabetes, medical institutions can recommend additional diagnostic tests and monitoring. If follow-up results confirm that the individual is not at risk, medical intervention can cease, allowing the person to continue with their life as usual. This approach balances the cost of unnecessary interventions with the benefit of reducing long-term healthcare expenses.

Medical institutions rely on early screening strategies developed by professionals to lower the prevalence of diabetes through preventive measures. Regular screening and health education raise public awareness of diabetes and promote healthier lifestyles, thereby reducing risk factors. Early interventions such as dietary adjustments, increased physical activity, and regular monitoring of blood glucose levels have been shown to effectively prevent the onset and progression of diabetes, aligning with global healthcare recommendations.

A critical challenge in developing predictive models for diabetes is the high imbalance commonly observed in medical datasets. Without proper handling, such imbalance can significantly degrade the performance of classification algorithms, as highlighted in previous studies [30]. To address this, various data preprocessing methods have been proposed, including oversampling, undersampling, and hybrid techniques. However, real-world applications often face challenges such as managing noise, scaling algorithms to large datasets, and preserving data quality. Transfer learning and active learning methods have been increasingly recommended as effective solutions for mitigating imbalanced data issues in classification tasks [31]. Nonetheless, imbalanced classification remains a well-recognized limitation for machine learning models, especially when the class distribution is severely skewed. This challenge persists despite recent advances in preprocessing and model design.

Transfer learning offers advantages such as data augmentation and dynamic update mechanisms, which improve model adaptability and performance in the target domain. However, it also poses challenges such as domain shift and the risk of negative transfer, which can reduce its effectiveness in certain cases. Active learning, on the other hand, excels in selectively labeling the most informative samples, thereby minimizing labeling costs and improving the classification of minority classes. Nonetheless, active learning methods face practical challenges, including sample selection bias and the relatively high cost of labeling samples in real-world applications.

In this study, we employed an active learning-based prediction approach during the model training phase, addressing the limitations associated with imbalanced datasets. By integrating SHAP for feature importance analysis and incorporating an attention mechanism, the proposed method achieved higher classification performance while requiring fewer labeled samples. This not only reduced the overall labeling cost but also enhanced the model’s ability to identify high-risk individuals accurately. The conclusions of this study are consistent with the findings of Lesci et al. [32], who demonstrated that active learning approaches improve model performance in tasks involving imbalanced datasets.

To ensure the observed improvements were statistically significant, we performed paired t-tests between the proposed model and baseline classifiers across the five folds of cross-validation. The improvements in F1-score and recall were statistically significant (p < 0.05), confirming the robustness of our approach.

We further examined misclassified cases and found that most false negatives (diabetic patients classified as non-diabetic) occurred in patients with borderline HbA1c and glucose levels. This suggests that incorporating additional longitudinal or lifestyle features may further reduce false negatives, which are particularly critical in clinical screening.

Our results further highlight the practical value of using active learning and SHAP-based methods in real-world clinical applications. By improving the efficiency of sample selection and focusing on the most critical features, the proposed approach ensures cost-effectiveness in resource-constrained settings. Furthermore, the model exhibited high recall, particularly for the minority class (diabetic patients), demonstrating its utility in early screening where identifying true positives is more crucial than avoiding false positives.

Additionally, the model demonstrated the ability to generalize across two distinct datasets, confirming its robustness and establishing it as a promising tool for early diabetes screening and intervention. While this study primarily adopted a 70/30 train-test split, future research could explore the effects of varying feature subsets and alternative split ratios to further assess the model’s robustness under diverse data conditions.

In addition, since K-Means clustering assumes spherical clusters and is sensitive to outliers, which may be suboptimal for medical datasets with complex nonlinear feature interactions, future research may consider clustering methods such as DBSCAN and Gaussian Mixture Models (GMM) as alternatives to better handle nonlinearity and noise.

These enhancements can be combined with domain adaptation techniques to address challenges related to dataset heterogeneity and further improve the scalability of active learning methods in large-scale clinical applications.

## Conclusion

We proposed a diabetes risk prediction model based on active learning. In this study, we used a dataset consisting of 100,000 data samples, of which 91,500 were classified as non-diabetic and 8,500 as diabetic. The evaluation metrics for the model included accuracy, precision, recall, and F1-score. Because the diabete risk prediction model outputs the classification variable, that is, whether there is a risk of diabetes, we introduce the confusion matrix to make the results more intuitive and applicable. Various machine learning classification algorithms were applied for diabetes risk prediction.

The challenge of handling imbalanced datasets lies in the natural scarcity of minority class samples and the potential for limited sample features. The dataset used in this study fits this scenario. Our primary objective was to demonstrate that using a prediction method based on active learning strategies can improve the performance of classification algorithms, particularly in addressing issues such as poor performance on minority classes, low recall, and weak generalization ability. By using focal active learning, we selected foci and dynamically updated the sub-pool to promote class balance and discover new clusters of minority class instances.

In the subsequent process, SHAP values were introduced to calculate feature importance, alongside an attention mechanism. SHAP values help identify features that significantly contribute to model predictions, allowing us to focus on these important features when selecting samples. This helps to avoid sample selection bias caused by relying solely on uncertainty or other single metrics, thereby enhancing the model’s transparency and interpretability. The attention mechanism adjusts feature weights based on their importance, enabling the model to focus more on critical features during sample selection, thus improving the model’s effectiveness and accuracy.

Throughout the process, feature weights were dynamically adjusted, allowing more reasonable sub-pool updates across different iterations and preventing overfitting to the initial decision boundaries. During the training phase, we compared the performance of different machine learning classification algorithms using evaluation metrics and benchmarked them against algorithms from recent studies. The classification reliability for diabetes risk prediction was significantly improved, outperforming the results from the literature [6,8–10,12,17,23–28].

The results indicate that combining focal active learning with the LGBM method yielded the best results, achieving an accuracy of 97.41% and a recall of 94.70% on the Diabetes-Prediction-Analysis dataset, which was validated through 5-fold cross-validation. These metrics clearly outperform traditional models that typically achieve 95% accuracy and 92% recall, highlighting the superiority of our method in both precision and robustness. Additionally, the proposed method also performed well on the other public dataset, further validating the generalization capability of the model.
